# Evaluation of Lateral Spread of Transgene Expression following Subretinal AAV–Mediated Gene Delivery in Dogs

**DOI:** 10.1371/journal.pone.0060218

**Published:** 2013-04-03

**Authors:** Ashlee R. Bruewer, Freya M. Mowat, Joshua T. Bartoe, Sanford L. Boye, William W. Hauswirth, Simon M. Petersen-Jones

**Affiliations:** 1 Department of Small Animal Clinical Sciences, Michigan State University, East Lansing, Michigan, United States of America; 2 Department of Ophthalmology, University of Florida College of Medicine, Gainesville, Florida, United States of America; National Eye Institute, United States of America

## Abstract

Dog models with spontaneously occurring mutations in retinal dystrophy genes are an invaluable resource for preclinical development of retinal gene therapy. Adeno-associated virus (AAV) vectors have been most successful; to target the outer retina and RPE they are delivered by subretinal injection, causing a temporary retinal detachment with some potential for retinal morbidity. A recent reporter gene study using an AAV2/8 vector in dogs reported transgene expression beyond the boundary of the subretinal bleb. This could be a desirable feature which increases the area of retina treated while minimizing the retinal detachment and any associated morbidity. We performed a detailed study of the lateral spread of transgene expression beyond the subretinal injection site following subretinally delivered AAV vectors in normal dogs. Vectors expressed green fluorescent protein (GFP) using a small chicken beta-actin promoter. AAV2/2 (quadruple tyrosine to phenylalanine (Y-F) capsid mutant), self-complementary (sc) AAV2/8 (single Y-F capsid mutant) and a scAAV2/5 were used. We found that in all eyes GFP expression involved retina beyond the initial post-injection subretinal bleb boundary. In all eyes there was post-injection spread of the retinal detachment within the first 3 days post procedure and prior to retinal reattachment. In 11/16 eyes this accounted for the entire “lateral spread” of GFP expression while in 5/16 eyes a very slight extension of GFP expression beyond the final boundary of the subretinal bleb could be detected. All 3 AAV constructs induced GFP expression in the nerve fiber layer with spread to the optic nerve. Patients treated by subretinal injection should be monitored for possible expansion of the subretinal injection bleb prior to reattachment. Injections in the para-foveal region may expand to lead to a foveal detachment that may be undesirable. Cell-specific promoters may be required to limit spread of expressed transgene to the brain with these AAV serotypes.

## Introduction

Retinal dystrophies such as the genetically heterogenous conditions Leber congenital amaurosis (LCA) and retinitis pigmentosa (RP) are important causes of human vision loss (see reviews [1], [2]). Similar retinal degenerative conditions occur spontaneously in animal species including dogs and cats [3], [4]. The dog in particular has been important in preclinical gene therapy trials. For example dogs with a spontaneous mutation in the *Rpe65* gene were pivotal in the development of phase I/II clinical trial programs for treatment of patients with mutations in the *RPE65* gene, an important cause of LCA [5]. Several other spontaneously occurring dog models have been identified and include those with mutations in *BEST1*, *CNGB3*, *rhodopsin*, *RPGR*, *PDE6A* and *PDE6B* [6], [7], [8], [9], [10], [11]. Preclinical gene therapy trials using some of these additional dog models have also been reported [12], [13], [14], [15].

Recombinant adeno-associated viral (AAV) vectors have become a favored gene delivery vehicle for the treatment of retinal dystrophies in both pre-clinical and clinical trials [16], [17]. Modification of AAV vectors have been used to alter tissue tropism and also speed and strength of transgene expression. These include pseudotyped vectors where the genome of a serotype 2 AAV (AAV2) is packaged into the capsid of another AAV serotype such as serotypes 5 or 8 to give a AAV2/5 or AAV2/8 vector. More recent advances in vector technology to improve transgene expression include the use of self-complementary genome constructs and site-directed mutations to change individual amino acid residues within capsid proteins. Self-complementary constructs have a normal single stranded DNA genome but with two copies of the gene of interest, one being complementary to the other, that are separated by a mutated terminal repeat site. This enables the transgene cDNA to rapidly form a double-stranded DNA molecule within the target cell, overcoming one of the rate-limiting steps, resulting in faster transgene expression [18], [19], [20], [21]. Mutation of capsid tyrosine residues to phenylalanine helps to protect the viral particle from proteasome degradation [22]; therefore, reducing the number of viral particles that are degraded resulting in stronger transgene expression. In the retina, these amino acid substitutions also affect cellular tropism [23], [24].

Subretinal injection of AAV is used when transgene expression is required in the retinal pigment epithelium (RPE) or the photoreceptors. The subretinal injection creates a temporary bullous detachment, separating the photoreceptor outer segments from the RPE layer. Typically the subretinal injection bleb resolves over the following few days both in humans [25] and dogs [21]. Subretinal injection likely has some deleterious effects on the photoreceptors, with such effects conceivably being more severe in a retina already compromised by disease. In particular it has been suggested that detaching the fovea in *RPE65*-LCA patients undergoing retinal gene therapy treatment may be detrimental [26].

A recent study by Stieger et al. investigating subretinal injection of AAV2/8 expressing green fluorescent protein (GFP) in the dog showed there was spread of GFP expression to retina adjacent to the initial subretinal injection site as well as centrally along the optic nerve to the brain, with the suggestion of trans-synaptic transmission [27]. Transduction of adjacent retina could be a desirable feature of newer generation AAV vectors as transgene expression beyond the initial boundary of the subretinal bleb might avoid some of the deleterious effects of creating a retinal detachment while maximizing the beneficial gene therapy effects. For example in human patients lateral spread of transduction could allow subretinal injection in the parafoveal region to produce transduction of the foveal cells while circumventing the deleterious effects of inducing a foveal detachment.

We performed a detailed study of the lateral spread of transduction beyond the initial boundary of the subretinal injection bleb using three different new generation AAV vectors. This showed that the vast majority of reporter gene expression beyond the initial boundary of the subretinal bleb was due to a continued detachment of the retina as the bleb flattened out over the first 3 days post injection.

## Materials and Methods

### Ethics Statement

All procedures were in compliance with the ARVO statement for the Use of Animals in Ophthalmic and Vision Research and approved by the Michigan State University Institutional Animal Care and Use Committee (AUF number 05-11-106-00; Institutional NIH/PHS Animal Welfare Assurance number A3955-01).

### Vectors

Recombinant AAV vectors were manufactured and purified by previously described methods [23], [24], [28]. This included purification and concentration by column chromatography. Vector titer was determined by real time PCR and final aliquots were resuspended in balanced salt solution (BSS Alcon Laboratories, Forth Worth TX, USA) containing 0.014% Tween 20. Three different AAV vectors were used: a self-complementary AAV2/5 construct expressing humanized green fluorescent protein (hGFP) driven by the ubiquitous, truncated chimeric CMV-chicken beta actin promoter (smCBA) (subsequently referred to as scAAV5); an AAV2/2 with four surface exposed capsid tyrosine residues replaced with phenylalanine (Y272F, Y444F, Y500F and Y730F) expressing green fluorescent protein driven by smCBA (subsequently referred to as AAV2(quadY-F); and a self-complementary expressing AAV2/8 construct with a single capsid tyrosine to phenylalanine mutation (Y733F) expressing hGFP driven by smCBA (subsequently referred to as scAAV8(Y733F).

### Animals/Subretinal Injection/Fundic Imaging

Eight normal young adult male laboratory beagle dogs were used.

Subretinal injections of viral vector were performed as previously described except that a standard three-port 23 gauge vitrectomy was included which allows for greater control in creating the subretinal injection [21]. The left eye of each dog received a subretinal injection of the scAAV5 vector. The right eye of four dogs received AAV2(quadY-F) and the right eye of the other four dogs scAAV8(Y733F). The titer used was 5×10^11^ vg/ml, the injection volume was 250 µl and the injections were made in the tapetal region of the fundus.

To monitor retinal reattachment and to detect transgene expression, fundus images were taken once daily for ten days and on day 14 and 21 post injection using a Retcam II digital video fundus camera (Clarity Medical Systems, Pleasanton, CA). Both regular color images and images using the fluorescein angiography settings (to detect GFP expression) were captured.

### Analysis of Fundus Images to Monitor Post-injection Expansion of Subretinal Bleb Prior to Retinal Reattachment

Fundus images from the post-operative days were examined to detect any post-injection expansion of the subretinal bleb. Using Photoshop (Photoshop CS6, Adobe Systems Inc. San Jose, CA) images showing the initial boundary of the subretinal bleb immediately post-injection were overlain by the color images showing the final boundary of the expanded subretinal bleb. Using Photoshop the transparency of the image on the upper layer was reduced to ∼50% to allow both layers to be visualized simultaneously. The images were moved or rotated as necessary to ensure precise superimposition of the superficial retinal vasculature at all edges of the detached area. Both the area delineated by the initial boundary of the subretinal bleb immediately post-injection and the area delineated by the final boundary of the expanded subretinal bleb was measured on the composite image in pixels using ImageJ (NIH [29]) The difference in area was calculated as the percentage increase in area.

### Analysis of Fundus Photographs to Compare Area of GFP Expression with Final Boundary of the Expanded Subretinal Bleb

To assess if GFP expression was detectable beyond the expanded subretinal bleb, an image showing the boundary of GFP fluorescence was superimposed on an image showing the final boundary of the expanded subretinal bleb. Again care was taken to ensure accurate superimposition of the retinal blood vessels in both images. The appearance of any GFP expression beyond the final boundary of the expanded subretinal bleb was examined to see if it represented likely spread along the nerve fiber layer towards the optic nerve head or simple expansion of the area of GFP to the adjacent retina.

### Retinal Histology

Following euthanasia the globes were removed and fixed in 4% paraformaldehyde as previously described [21]. Eye cups and optic nerve cross sections were mounted in optimal cutting temperature medium (TissueTek OCT, Electron Microscopy Sciences, Hatfield PA) and frozen. 14 µm sagittal cryosections were cut using a cryomicrotome (Leica CM3050-S, Leica Microsystems, Buffalo Grove IL) onto poly-L-lysine coated glass slides (Electron Microscopy Sciences, Hatfield MO). Slides were dried for 20 minutes, rehydrated in PBS for 20 minutes, then blocked for 1 hour at room temperature using a solution of PBS containing 0.1% Triton X-100 (Sigma-Aldrich, St. Louis MO) and 10% normal horse serum (Sigma-Aldrich, St. Louis MO). They were stained using a rabbit FITC conjugated anti-GFP antibody (Invitrogen Corp, Carlsbad CA) at 1∶1000 overnight at 4°C. The nuclear counterstain DAPI (Invitrogen Corp, Carlsbad CA) was used. Slides were mounted using antifade fluorescent mounting medium (Dako North America Inc, Carpinteria CA) and glass coverslips (Electron Microscopy Sciences, Hatfield MO) and stored at 4°C in the dark until imaging.

### Statistical Analysis

A split –plot ANOVA was used to look for interaction between dog, eye injected, vector type, initial size of bleb, final size of bleb and percentage increase in bleb size (SAS. SAS Institute, Cary North Carolina). Significance was considered at *p*<0.05.

## Results

### Post-injection Expansion of Subretinal Bleb

**Figure 1:**
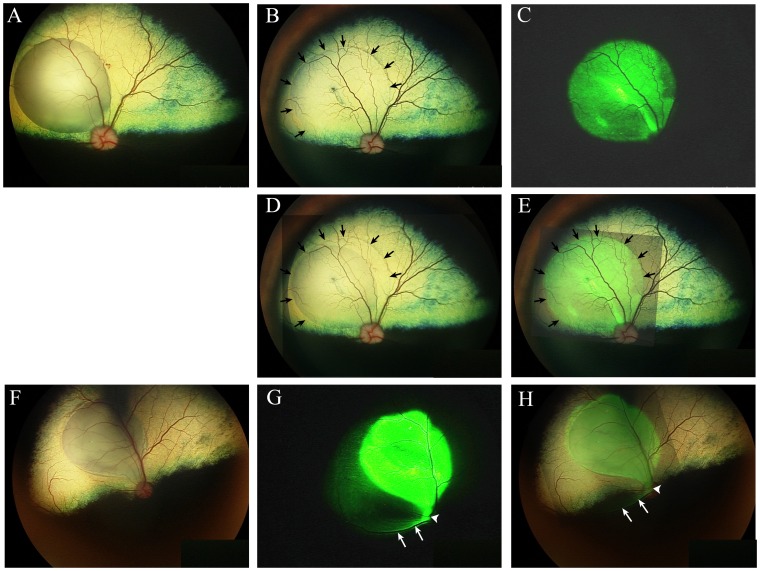
Color and fluorescent fundus images. A-E. Eye 7 OD (injected with scAAV8(Y733F)). F-G dog 5 OD (injected with scAAV5). A. The subretinal bleb of eye 7 OD immediately post-injection. B. The same eye as in A with arrows indicating the final boundary of the expanded subretinal bleb which is clearly visible in the fundus image. C. The same eye as in A showing in vivo GFP expression. D. A composite of image A and B to show expansion of the subretinal bleb prior to retinal reattachment (arrows are the same as in B). Note that the final boundary of the expanded subretinal bleb well beyond the initial boundary of the subretinal bleb immediately post-injection (an increase of 14.7% in size). E. A composite of images B and C. GFP expression is limited to the area delineated by the final boundary of the expanded subretinal bleb. F. The subretinal bleb of eye 5 OD immediately post-injection. G. The same eye as in F showing in vivo GFP expression. Arrows indicate regions of suspected nerve fiber layer GFP expression. The arrowhead indicates optic nerve head GFP expression. Note the retinal region medial to the subretinal bleb shows a pattern suggestive of GFP fluorescence within ganglion cell axons. Arrows indicate some of the stronger GFP expression in presumptive nerve fiber layer and the arrowhead indicates the GFP expression involving the optic nerve head. H. An overlay of images F and G. There is slight spread of GFP beyond the post-injection extent of the bleb. Arrows and arrowhead are as described for G.

In all 16 eyes the subretinal injection caused a focal area of bullous retinal detachment (Figure 1a). This subretinal bleb flattened over the next few days with retinal reattachment occurring in all cases. Comparison of the immediate post-injection fundus image with the fundus images captured over the first four days post-injection showed that in all of the eyes there was further expansion of the subretinal bleb increasing the area of retina detached prior to eventual retinal reattachment (Figure 1A, B & D. Table 1). There was no further expansion of the subretinal bleb after the first day in 7 of 16 eyes, the second day in 7 of 16 eyes and the third day in 2 of 16 eyes (a mean of 1.7 days). There was no difference in the day at which the final boundary of the expanded subretinal bleb was achieved between the three vector types (a mean of 1.625 days for scAAV5 and 1.75 days for both AAV2(quadY-F) and scAAV8(Y733F). The increase in area delineated by the final boundary of the expanded subretinal bleb compared to the area delineated by the initial boundary of the subretinal bleb immediately post-injection and the area as measured from composite images ranged between 2.6% and 37.4%. The degree of expansion of the subretinal bleb was not significantly different between the three vector types used and showed no correlation with initial area of the subretinal bleb (*p*>0.05. data not shown).

**Table 1:** Results of spread of injection bleb and extent of final GFP expression.

Vector	Dog (eye)	Days to finalsubretinal blebexpansion	Initial bleb area(measured in‘000 pixels)	% Increasein subretinalbleb size	GFP expressiondetected beyondfinal boundary ofsubretinal bleb	GFP expressiondetected in NFLor ONH
scAAV5	1 (OS)	1	97.0	27.1	0	ONH*
	2 (OS)	2	66.7	13.8	0	NFL
	3 (OS)	1	60.6	5.7	0	ONH*
	4 (OS)	2	45.8	15.0	0	ONH*
	5 (OS)	2	64.8	12.2	0	ONH; NFL
	6 (OS)	2	91.4	24.2	0	ONH*
	7 (OS)	2	79.9	6.6	0	ONH*
	8 (OS)	1	63.7	23.6	+	ONH*
	**Mean**	1.625	71.2	16.0		
AAV2(quadY-F)	1 (OD)	1	138.1	6.8	+	ONH*
	2 (OD)	3	52.7	23.5	+	ONH; NFL
	6 (OD)	2	87.1	37.4	0	ONH*
	8 (OD)	1	51.9	4.8	0	ONH; NFL
	**Mean**	1.75	82.5	18.1		
scAAV8(Y733F)	3 (OD)	1	72.3	17.9	+	ONH; NFL
	4 (OD)	2	110.4	2.6	+	ONH*
	5 (OD)	3	38.3	8.8	0	ONH*; NFL
	7 (OD)	1	53.6	14.7	0	ONH*
	**Mean**	1.75	68.6	11.0		

* - indicates that bleb contacted edge of ONH following injection.

Key: NFL = Nerve fiber layer; OD = right eye; OS = left eye; ONH = optic nerve head.

### GFP Expression Beyond the Expanded Subretinal Bleb

**Figure 2:**
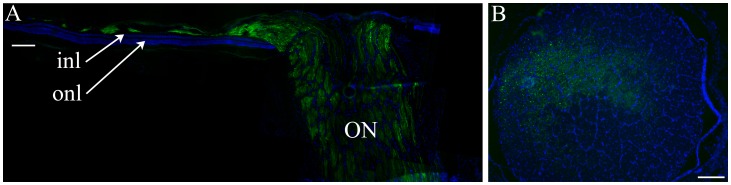
A. Low power retinal section from eye 8-OD through the optic nerve (ON). Green shows GFP expression and blue is DAPI staining. Note that there is GFP expression in axons within the optic nerve and also within the part of the inner retina corresponding to the nerve fiber layer. The section is taken through the dorsal vasculature radiating from the optic nerve head and the blood vessels are the reason that the nerve fiber layer is not a continuous layer in this section. This region is away from the subretinal injection bleb which is why only the nerve fiber layer is expressing GFP and the outer retina is not. Inl = inner nuclear layer; onl = outer nuclear layer. Size bar 100 µm. B. Section through the optic nerve from eye 2-OD. This eye received an injection of AAV2(quadY-F). Green is GFP expression, blue DAPI staining. GFP expression is seen in the axons of the optic nerve. Size bar 250 µm.

In 15 out of 16 eyes *in-vivo* fluorescence of the optic nerve head was apparent once GFP expression in the subretinal bleb became well established (Figure 1G and H). Subsequently on histological examination GFP expression was seen in the nerve fiber layer (Figure 2A) and sections of the optic nerve from all eyes (Figure 2A and B). In 11 of the 15 eyes with detectable *in-vivo* fluorescence of the optic nerve head the subretinal bleb contacted the edge of the nerve head. In the four eyes where the bleb did not contact the edge of the optic nerve head GFP expression extended past the final boundary of the expanded subretinal bleb to the optic nerve head in a pattern that suggested it was within the nerve fiber layer (this was confirmed histologically and a representative image is shown in Figure 2). In one eye (2 OS) GFP expression extended past the final boundary of the expanded subretinal bleb in a similar pattern suggestive that it was in the nerve fiber layer; however, in this eye no GFP-expression was detected in optic nerve head in fundic images, although subsequently GFP expression was detected in the optic nerve by histology. In one eye (5 OD), there was obvious involvement of the nerve fiber layer in addition to direct contact of the subretinal bleb with the edge of the optic nerve head (Figure 1F–H).

With the exception of the aforementioned involvement of the nerve fiber layer, GFP expression was isolated to the area delineated by the final boundary of the expanded subretinal bleb in 11 of the 16 eyes.

**Figure 3:**
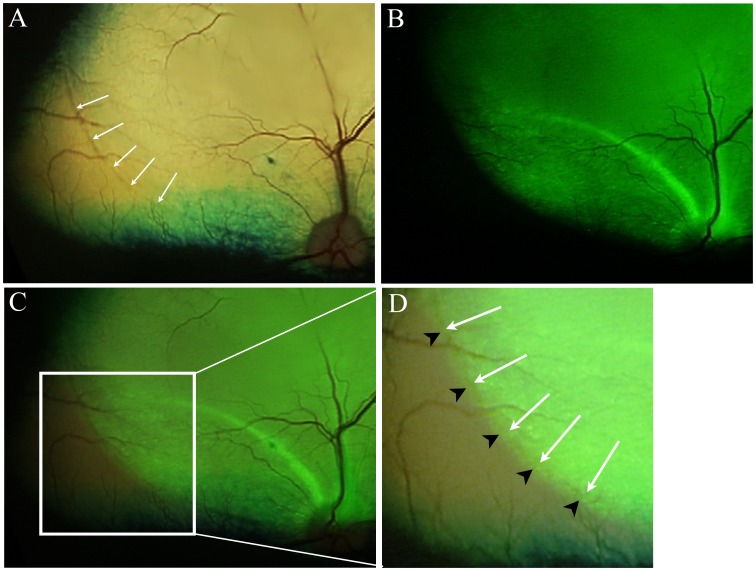
Expression of GFP beyond the final extent of the retinal detachment. Dog 1 OD – this eye received an injection of AAV2(quadY-F). A. Color fundus image following retinal reattachment after the subretinal injection. The final extent of the detachment can be seen as a color change in the tapetal fundus as indicated by arrows. B. The same eye imaged to show GFP expression. C. An overlay of the fundus images in A and B. D. An enlarged view of the region in C that is within the white box. The white arrows correspond to the arrows in A. The black arrow heads point out the extent of the GFP expression which extends slightly beyond the final boundary of the retinal detachment.

In 5 eyes (Table 1, Figure 3) careful examination of the composite color and fluorescent fundus images showed that there was GFP expression that extended slightly beyond the final boundary of the expanded subretinal bleb unrelated to apparent nerve fiber layer GFP expression. The extent of GFP expression beyond the final boundary of the expanded subretinal bleb measured less than one quarter of the optic nerve head diameter in all cases (the optic nerve head in dogs is ∼2mm in diameter). This extension of GFP expression was detected in one of the scAAV5 injected eyes, 2 of the AAV2(quadY-F) injected eyes, and 2 of the scAAV8(Y733F) injected eyes.

## Discussion and Conclusions

We show in this study that retinal GFP expression extends beyond the boundary of the subretinal bleb immediately post-injection. This supports the findings of Stieger et al. [27] (see their Figure 2 a, b, c) describing retinal transduction beyond the original subretinal bleb using an AAV2/8 vector. However, we questioned how much of this extension to the adjacent retina was due to lateral spread of vector through an intact retina and how much was due to prolonged expansion of the subretinal bleb. We found by closely monitoring the treated eyes over the first ten days post-injection that the subretinal bleb expanded in all eyes before the retina finally reattached. A large proportion of the GFP expression beyond the boundary of the subretinal bleb immediately post-injection was accounted for by this expansion of the subretinal bleb. This expansion was variable and in some eyes was quite considerable, resulting in an area delineated by the final boundary of the expanded subretinal bleb between 102.6% and 137.4% of the area delineated by the initial boundary of the subretinal bleb immediately post-injection. The degree of bleb expansion did not correlate with either the area delineated by the initial boundary of the subretinal bleb immediately post-injection or with the vector serotype. In some dogs the degree of bleb expansion differed considerably between left and right eye suggesting that individual variability between dogs was not on strong influence on outcome. The same injection volume was used for each eye in this study but factors such as the volume of vector refluxed through the retinotomy and the post-operative intraocular pressure may have affected the tendency for the bleb to expand. The duration over which the subretinal bleb expansion occurred did differ between eyes; however, after the third post-operative day no further expansion was detected in any of the 16 eyes.

In 5 of the 16 we noted GFP expression beyond the final boundary of the expanded subretinal bleb on careful comparison of *in-vivo* fluorescent fundus images with the color images. This extension was very slight, but suggests some lateral spread of vector through the intact retina either by spread though extracellular spaces or even across cells themselves. In separate studies with AAV we have shown transretinal passage of new generation AAV vectors can occur in the dog, with outer retinal reporter transgene expression occurring following intravitreal administration of vectors [30].

A second pattern of GFP expression beyond the subretinal bleb was also detected. The pattern of this GFP expression suggested transit within the nerve fiber layer. In 15 of the 16 eyes *in-vivo* fluorescent fundus imaging showed GFP expression within the optic nerve head and on histological sections GFP was detected in ganglion cells and in all 16 optic nerves. Histology also confirmed that GFP expression occurred in the nerve fiber layer and passed over areas in which no GFP expression was detectable (see Figure 2). Potential trans-synaptic spread of AAV8 following subretinal injection in the dog was suggested by Stieger et al. [27]. In this study we also found that the scAAV5 and the AAV2(quadY-F) vectors (in addition to AAV2/8) transduced the axons of ganglion cells following subretinal injection. The vector delivery in this study and that of Stieger et al study was by subretinal injection using a transvitreal approach. This procedure requires the injection into the subretinal space to be made through a small retinotomy. Reflux of vector into the vitreous may occur through the retinotomy post-injection and some leakage into the vitreous at the time of injection could also occur if the injection cannula does not seal completely with the retinotomy. Thus exposure of ganglion cells to vector within the vitreous rather than spread to the ganglion cells trans-synaptically from the subretinal space is possible. Future studies using a transcleral approach to subretinal injection [31] that avoids leakage of vector into the vitreous could be used to determine if transgene expression in the nerve fiber layer occurs as a result of transretinal spread emanating from the subretinal bleb, or from vector that may have leaked into the vitreous.

There are a number of important findings from this study. First we found there is progression of the subretinal bleb for up to 3 days following a subretinal injection in the dog. If similar expansion of the subretinal bleb occurs in human patients, injections made to treat parafoveal photoreceptors could lead to post-operative expansion of the detachment to involve the fovea. As it has been suggested foveal detachments from subretinal injections may have deleterious effects [26] vitreoretinal surgeons performing subretinal injection in patients should monitor subretinal blebs carefully until complete reattachment occurs. Second, in addition to AAV8 vectors, new generation AAV2 and AAV5 vectors can also transduce ganglion cells further raising the concern for off-target spread of gene therapy vectors to the brain. Use of tissue specific promoters may be required to carefully control which cell types express the delivered transgenes. Detailed investigation of the cell types transduced by the vectors reported in this study will form part of a separate report.

## References

[1] Berson EL (2008) Retinal degenerations: planning for the future. Adv Exp Med Biol 613: 21–35. 18188925 10.1007/978-0-387-74904-4_2

[2] Stone EM (2007) Leber congenital amaurosis - a model for efficient genetic testing of heterogeneous disorders: LXIV Edward Jackson Memorial Lecture. Am J Ophthalmol 144: 791–811. 17964524 10.1016/j.ajo.2007.08.022

[3] Beltran WA (2009) The use of canine models of inherited retinal degeneration to test novel therapeutic approaches. Vet Ophthalmol 12: 192–204. 19392879 10.1111/j.1463-5224.2009.00694.x PMC3193984

[4] Baehr W, Frederick JM (2009) Naturally occurring animal models with outer retina phenotypes. Vision Res 49: 2636–2652. 19375447 10.1016/j.visres.2009.04.008 PMC2782402

[5] Acland GM, Aguirre GD, Ray J, Zhang Q, Aleman TS, et al (2001) Gene therapy restores vision in a canine model of childhood blindness. Nat genet 28: 92–95. 11326284 10.1038/ng0501-92

[6] Guziewicz KE, Zangerl B, Lindauer SJ, Mullins RF, Sandmeyer LS, et al (2007) Bestrophin gene mutations cause canine multifocal retinopathy: a novel animal model for best disease. Invest Ophthalmol Vis Sci 48: 1959–1967. 17460247 10.1167/iovs.06-1374 PMC1931491

[7] Sidjanin DJ, Lowe JK, McElwee JL, Milne BS, Phippen TM, et al (2002) Canine CNGB3 mutations establish cone degeneration as orthologous to the human achromatopsia locus ACHM3. Hum Mol Genet 11: 1823–1833. 12140185 10.1093/hmg/11.16.1823

[8] Kijas JW, Cideciyan AV, Aleman TS, Pianta MJ, Pearce-Kelling SE, et al (2002) Naturally occurring rhodopsin mutation in the dog causes retinal dysfunction and degeneration mimicking human dominant retinitis pigmentosa. Proc Natl Acad Sci USA 99: 6328–6333. 11972042 10.1073/pnas.082714499 PMC122948

[9] Beltran WA, Hammond P, Acland GM, Aguirre GD (2006) A Frameshift Mutation in RPGR Exon ORF15 Causes Photoreceptor Degeneration and Inner Retina Remodeling in a Model of X-Linked Retinitis Pigmentosa. Investigative Ophthalmology and Visual Science 47: 1669–1681. 16565408 10.1167/iovs.05-0845

[10] Tuntivanich N, Pittler SJ, Fischer AJ, Omar G, Kiupel M, et al (2009) Characterization of a canine model of autosomal recessive retinitis pigmentosa due to a PDE6A mutation. Invest Ophthalmol Vis Sci 50: 801–813. 18775863 10.1167/iovs.08-2562 PMC3720143

[11] Suber ML, Pittler SJ, Quin N, Wright GC, Holcombe N, et al (1993) Irish setter dogs affected with rod-cone dysplasia contain a nonsense mutation in the rod cGMP phosphodiesterase beta-subunit gene. Proc Natl Acad Sci USA 90: 3968–3972. 8387203 10.1073/pnas.90.9.3968 PMC46427

[12] Komaromy AM, Alexander JJ, Rowlan JS, Garcia MM, Chiodo VA, et al (2010) Gene therapy rescues cone function in congenital achromatopsia. Hum Mol Genet 19: 2581–2593. 20378608 10.1093/hmg/ddq136 PMC2883338

[13] Beltran WA, Cideciyan AV, Lewin AS, Iwabe S, Khanna H, et al (2012) Gene therapy rescues photoreceptor blindness in dogs and paves the way for treating human X-linked retinitis pigmentosa. Proc Natl Acad Sci USA 109: 2132–2137. 22308428 10.1073/pnas.1118847109 PMC3277562

[14] Mowat FM, Bartoe JT, Bruewer A, Dinculescu A, Boye SL, et al (2012) Evaluation Of Rod Photoreceptor Function And Preservation Following Retinal Gene Therapy In The PDE6A Mutant Dog. ARVO Meeting Abstracts 53: 1928.

[15] Petit L, Lheriteau E, Weber M, Le Meur G, Deschamps JY, et al (2012) Restoration of Vision in the pde6beta-deficient Dog, a Large Animal Model of Rod-cone Dystrophy. Mol Ther 20: 2019–2030. 22828504 10.1038/mt.2012.134 PMC3498794

[16] Auricchio A, Rolling F (2005) Adeno-associated viral vectors for retinal gene transfer and treatment of retinal diseases. Curr Gene Ther 5: 339–348. 15975011 10.2174/1566523054065020

[17] Buch PK, Bainbridge JW, Ali RR (2008) AAV-mediated gene therapy for retinal disorders: from mouse to man. Gene Ther 15: 849–857. 18418417 10.1038/gt.2008.66

[18] McCarty DM, Fu H, Monahan PE, Toulson CE, Naik P, et al (2003) Adeno-associated virus terminal repeat (TR) mutant generates self-complementary vectors to overcome the rate-limiting step to transduction in vivo. Gene Ther 10: 2112–2118. 14625565 10.1038/sj.gt.3302134

[19] McCarty DM (2008) Self-complementary AAV vectors; advances and applications. Mol Ther 16: 1648–1656. 18682697 10.1038/mt.2008.171

[20] Natkunarajah M, Trittibach P, McIntosh J, Duran Y, Barker SE, et al (2008) Assessment of ocular transduction using single-stranded and self-complementary recombinant adeno-associated virus serotype 2/8. Gene Ther 15: 463–467. 18004402 10.1038/sj.gt.3303074

[21] Petersen-Jones SM, Bartoe JT, Fischer AJ, Scott M, Boye SL, et al (2009) AAV retinal transduction in a large animal model species: comparison of a self-complementary AAV2/5 with a single-stranded AAV2/5 vector. Mol Vis 15: 1835–1842. 19756181 PMC2743804

[22] Zhong L, Li B, Mah CS, Govindasamy L, Agbandje-McKenna M, et al (2008) Next generation of adeno-associated virus 2 vectors: point mutations in tyrosines lead to high-efficiency transduction at lower doses. Proc Natl Acad Sci USA 105: 7827–7832. 18511559 10.1073/pnas.0802866105 PMC2402387

[23] Petrs-Silva H, Dinculescu A, Li Q, Min SH, Chiodo V, et al (2009) High-efficiency transduction of the mouse retina by tyrosine-mutant AAV serotype vectors. Mol Ther 17: 463–471. 19066593 10.1038/mt.2008.269 PMC2835095

[24] Petrs-Silva H, Dinculescu A, Li Q, Deng WT, Pang JJ, et al (2011) Novel properties of tyrosine-mutant AAV2 vectors in the mouse retina. Mol Ther 19: 293–301. 21045809 10.1038/mt.2010.234 PMC3034844

[25] Bainbridge JW, Smith AJ, Barker SS, Robbie S, Henderson R, et al (2008) Effect of gene therapy on visual function in Leber’s congenital amaurosis. N Engl J Med 358: 2231–2239. 18441371 10.1056/NEJMoa0802268

[26] Jacobson SG, Cideciyan AV, Ratnakaram R, Heon E, Schwartz SB, et al (2012) Gene therapy for leber congenital amaurosis caused by RPE65 mutations: safety and efficacy in 15 children and adults followed up to 3 years. Arch Ophthalmol 130: 9–24. 21911650 10.1001/archophthalmol.2011.298 PMC3600816

[27] Stieger K, Colle MA, Dubreil L, Mendes-Madeira A, Weber M, et al (2008) Subretinal delivery of recombinant AAV serotype 8 vector in dogs results in gene transfer to neurons in the brain. Mol Ther 16: 916–923. 18388922 10.1038/mt.2008.41

[28] Hauswirth WW, Lewin AS, Zolotukhin S, Muzyczka N (2000) Production and purification of recombinant adeno-associated virus. Methods Enzymol 316: 743–761. 10800712 10.1016/s0076-6879(00)16760-6

[29] Schneider CA, Rasband WS, Eliceiri KW (2012) NIH Image to ImageJ: 25 years of image analysis. Nat Methods 9: 671–675. 22930834 10.1038/nmeth.2089 PMC5554542

[30] Bartoe JT, Mowat F, Dinculescu A, Boye S, Hauswirth W, et al (2011) AAV Vectors With Engineered Capsid Mutations Efficiently Transduce Outer Retina With Intravitreal Delivery And Result In Rapid Gene Expression With Subretinal Delivery In Dogs. ARVO Meeting Abstracts 52: 1420.

[31] Verdugo ME, Ailing J, Lazar ES, del Cerro M, Ray J, et al (2001) Posterior segment approach for subretinal transplantation or injection in the canine model. Cell Transplant 10: 317–327. 11437077

